# PEGylated thymosin β4 is a thiol‐site‐specific prodrug treating myocardial infarction in vivo

**DOI:** 10.1002/btm2.70144

**Published:** 2026-03-31

**Authors:** Haisheng Peng, Yanqun Chai, Chen Gong, Yue Hao, Yanbo Peng, Wenyuan Zhang, Xiaoying Liu, Shukun Tang, Qingfei Kong, Qun Wang

**Affiliations:** ^1^ Department of Pharmacology, Medical College University of Shaoxing Shaoxing China; ^2^ Department of Pharmacy, Daqing Branch Harbin Medical University Daqing China; ^3^ Department of Neurobiology Harbin Medical University, Heilongjiang Provincial Key Laboratory of Neurobiology Harbin Heilongjiang China; ^4^ Department of Pharmaceutical Engineering China Pharmaceutical University Nanjing China; ^5^ Department of Chemical and Biological Engineering Iowa State University Ames Iowa USA

**Keywords:** apoptosis, myocardial infarction, PEGylation, prodrug, thymosin β4

## Abstract

Thymosin beta 4 (Tβ_4_) has been clinically trialed for over 10 years to treat ulcers, dry eye syndrome, and acute myocardial infarction (MI). However, as of now, no Tβ4 drug has been approved. Tβ_4_, as a small protein drug, has to face druggability challenges such as abundant supply, high purity, verified efficacy and safety, long half‐life, and shelf‐life. Here, a modified prokaryotic expression system was developed to express recombination Tβ_4_ (rTβ_4_), followed by single thiol‐site‐specific PEGylation of rTβ_4_, namely PEG‐rTβ_4_ as a prodrug. The identification and thermodynamic properties of PEG‐rTβ_4_ were tested with a matrix‐assisted laser desorption‐ionization time of flight mass spectrometer, a differential scanning calorimeter, and a thermal gravimetric analyzer. The data showed the superiority of PEG‐rTβ_4_ for treating MI. Long‐circulating PEG‐rTβ4 significantly relieves myocardial remodeling, restores cardiac function, promotes neoangiogenesis, and inhibits apoptosis via the Akt/Bcl‐2/caspase‐3 pathway. In summary, PEG‐rTβ_4_ is a better choice for the drug development of this active protein.

## Graphical abstract

The synthesis and schematic structure of PEGylated recombination thymosin β‐4 (PEG‐rTβ_4_) and the illustration of targeted therapy for myocardial infarction (MI) in rats.

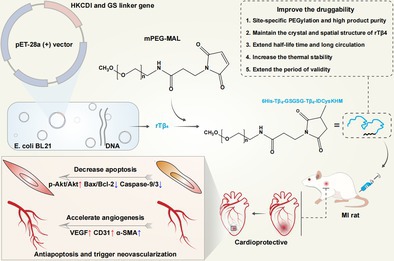

**Translational Impact Statements**

We developed PEGylation of recombinant Tβ4 (rTβ4), namely PEG‐rTβ4, as a prodrug for treating myocardial infarction and evaluated its efficacy in rats. PEG‐rTβ4 can significantly inhibit myocardial remodeling, elevate VEGF levels, and further increase artery and capillary density in the infarct heart. We confirmed that PEG‐rTβ_4_ accelerated angiogenesis via the Akt/Bcl‐2/caspase‐3 pathway, making it a better choice for drug development of this active protein for clinical applications.

**Abbreviations**
CD31: platelet endothelial cell adhesion molecule 1 (PECAM 1)
DSC: differential scanning calorimetry
ECG: electrocardiograph
EF: ejection fraction
HE: hematoxylin–eosin
I‐KI: iodine‐potassium iodide
LAD: left anterior‐descending artery
LVEDD: left ventricular end‐diastolic diameter
LVEDV: left ventricular end‐diastolic volume
LVEF: left ventricular ejection fraction
LVESD: left ventricular end‐systolic diameter
LVESV: left ventricular end‐systolic volume
LY: LY294002
MAL: maleinimide
MALDI‐TOF MS: matrix‐assisted laser desorption‐ionization time of flight mass spectrometry
MI: myocardial infarction
PBS: phosphate‐buffered saline
PEG: polyethylene glycol
PEG‐MAL: polyethylene glycol 2000 with maleinimide
PEG‐rTβ_4_
: PEGylated rTβ_4_
PVDF: polyvinylidene fluoride
rTβ_4_
: recombination thymosin β‐4
SDS‐PAGE: sodium dodecyl sulfonate polyacrylamide gel electrophoresis
TCEP: trichloroethyl phosphate
TGA: thermal gravimetric analysis
TTC: triphenyl tetrazolium chloride
Tβ_4_
: thymosin β‐4
VEGF: vascular endothelial growth factor
α‐SMA: α‐smooth muscle actin

## 1 INTRODUCTION

Cardiovascular diseases (CVD) are common lethal disorders with high incidence worldwide and will lead to 23 million deaths by 2030. Myocardial infarction (MI) is the most common syndrome of CVD, causing pathological remodeling and heart failure (HF).^1^ The development of cardiovascular drugs has become a vital field of the pharmaceutical industry. Thymosin beta 4 (Tβ_4_), a 43‐amino acid peptide isolated from thymus in the 1980s, has a molecular weight of 4921 Da (4963 Da acetylated).^2^, ^3^ It regulates actin by binding free actin monomers in a 1:1 ratio, maintaining the equilibrium between fibrous and spherical actin, thereby stimulating cell movement and regulating proliferation, differentiation, and apoptosis.^4^ Tβ_4_ is critical in wound healing, tissue regeneration, angiogenesis, tumorigenesis, and hair follicle development.^5^, ^6^, ^7^, ^8^ Recent studies focus on its role in acute MI, where it reduces cell apoptosis and inflammation, and in chronic cases, it promotes capillary growth and pericyte recruitment while inhibiting fibrosis.^9^ The druggability of Tβ_4_ has been explored for decades, with 17 clinical studies since 2008, 11 completed,^10^ but no Tβ_4_ formulation has been approved for marketing.

The translation of Tβ_4_ faces many challenges, as follows: First, the industry requires a sufficient supply of active protein, which is achieved through gene recombination to replace traditional methods due to low animal tissue content and yield. Tβ_4_'s small size complicates its expression and purification, necessitating a better solution.^11^, ^12^, ^13^ The advancement of biochemistry and molecular biology has led to techniques for optimizing Tβ4 expression, such as gene recombination and codon optimization.^14^ Second, scientists face other challenges in the druggability of Tβ_4_, including dosage design, efficacy, safety, half‐life, and shelf‐life. A Phase 1 safety study of an intravenous (IV) administration of Tβ_4_ in healthy volunteers showed that increasing the Tβ_4_ dose from 42 to 1260 mg only raised its half‐life from 0.95 to 2.1 h (Phase 2, NCT05485818, 2021).^15^ The therapeutic concentration for acute MI is 250–2000 ng/kg, later narrowed to 500–1500 ng/kg to enhance efficacy and reduce side effects (phase 2, NCT 05984134, 2023). Third, polymeric prodrug strategies can improve the druggability of active pharmaceutical ingredients,^16^, ^17^, ^18^ yet research on polymer‐modified Tβ_4_ is lacking. Polyethylene glycol (PEG) modification (PEGylation) can improve the solubility and mobility of proteins or peptides in solution, enhance their stability in blood, and reduce their toxicity, immunogenicity, and antigenicity, overcoming critical barriers to these macromolecules in the body.^19^ In addition, it can prolong the half‐life, alter the distribution, and regulate the release of active units from PEGylated conjugates.^20^ So far, several PEGylated drugs have been approved by the US Food and Drug Administration.^21^, ^22^, ^23^ Therefore, PEGylation may address the druggability issues of Tβ_4_. Although PEGylation of this protein has many benefits, the indeterminacy of the conjugation site may lead to decreased purity of the raw protein. This is especially true because the reaction involves conjugation at amino and carboxyl groups within the protein chain, which complicates the purification process.^24^ Fourth, although the applications of Tβ_4_ due to the features of anti‐apoptosis and neovascularization could be turned into reality,^25^, ^26^, ^27^ the effects of PEGylation on the signal regulation of Tβ_4_ also remain unknown.

**FIGURE 1:**
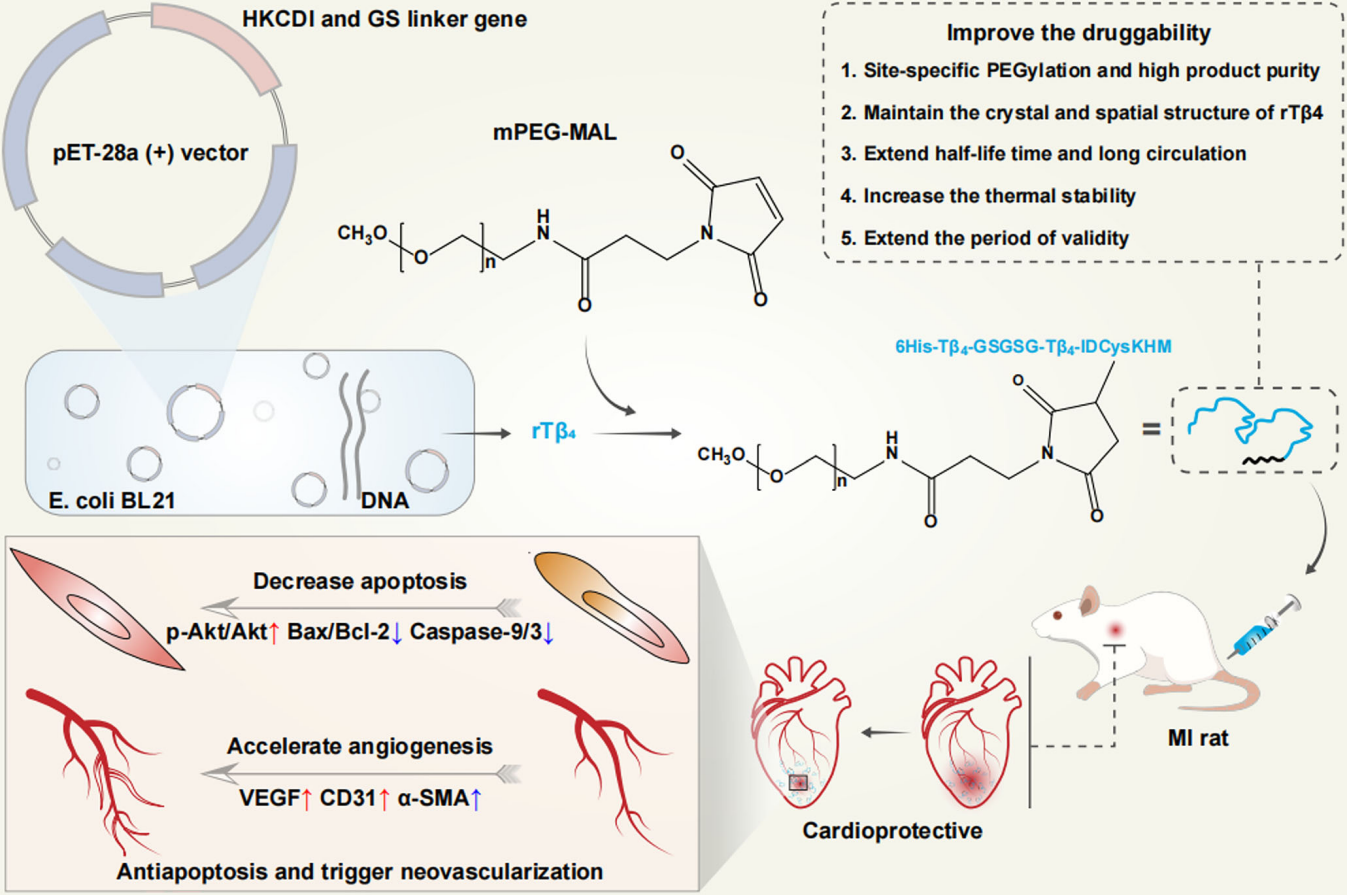
Schematic structure and the application of PEGylated recombination thymosin β‐4 (PEG‐rTβ_4_) in myocardial infarction (MI). PEG‐MAL, polyethylene glycol 2000 with maleinimide; VEGF, vascular endothelial growth factor; α‐SMA, α‐smooth muscle actin.

We designed a long‐circulating prodrug of recombinant Tβ_4_ (rTβ_4_), PEGylated rTβ_4_ (PEG‐rTβ_4_). This prodrug has two rTβ_4_ molecules coupled with maleimide‐functionalized PEG_2000_ at a single cysteine (Cys) thiol site, as shown in Figure 1. The gene recombination technology was used to overcome the deficiency of Tβ_4_ in preparation. While a site‐specifically PEG‐rTβ_4_ has improved properties, including higher purity, better physical and thermal stability, and a longer circulating half‐life in vivo, we consider that these properties of PEG‐rTβ4 have led to better therapeutic effects in MI.

## 2 MATERIALS AND METHODS

### 2.1 Materials

*Escherichia coli* (*E. coli*) BL21 (DE3) Chemically Competent Cell was provided by TransGen Biotech, Co., Ltd. (Peking, China). Recombinant thymosin β‐4 (rTβ_4_) whole‐length gene codon synthetic and cloned into expression vector pET‐28a was completed by Suzhou Hongxun Biotechnology Co., Ltd. (Suzhou, China). Polyethylene glycol 2000 with maleinimide (PEG‐MAL) (mPEG_2000_‐(CH_2_)_3_ NHCO (CH_2_)_2_‐MAL) was bought from Xi'an Ruixi Biological Technology Co., Ltd. (Xi'an, China). Trichloroethyl phosphate (TCEP) and LY294002 (LY, Akt inhibitor) were purchased from Shanghai Duma Biotechnology Co., Ltd. (Shanghai, China). 3,3‐Dioctadecyloxacarbocyanine perchlorate (DiO) and Hoechst 33258 were obtained from Peking Fanbo Science & Technology, Co., Ltd. (Peking, China). Histidine‐tagged antibody was purchased from Harbin Nachuan Biotechnology Co., Ltd. (Harbin, China). Antibody vascular endothelial growth factor (VEGF) and α‐smooth muscle actin (α‐SMA) were purchased from Peking Bioss Biotech, Co., Ltd. (Peking, China). Antibody against platelet endothelial cell adhesion molecule 1 (CD31) was obtained from Wuhan Huamei Bioengineering Co., Ltd. (Wuhan, China). Anti‐β‐actin, anti‐Akt, anti‐p‐Akt, anti‐Bax, anti‐Bcl‐2, anti‐cleaved caspase‐3, and anti‐cleaved caspase‐9 were bought from Abcam's Heilongjiang Regional agent in China. The JC‐1 kit for assessing mitochondrial membrane potential was provided by Peking Beyotime Biotechnology Co., Ltd. (Peking, China). Masson's Trichrome Staining Kit was supplied by Peking Solarbio Biotechnology (Peking, China). The public research platform in college provides other related reagents and materials.

### 2.2 Cells and animals

*E. coli* BL21 (DE3) Chemically Competent Cell (TransGen Biotech, Co., Ltd., Peking, China) was cultured in an Luria‐Bertani agar culture medium after the transfection of a recombinant plasmid, pET‐28a‐rTβ_4_. H9c2 cells were provided by the China Academia Sinica Cell Repository (Shanghai, China) and maintained in Dulbecco's Modified Eagle Medium with 10% fetal bovine serum at 37°C in a 5% CO_2_ atmosphere. Two‐day‐old Wistar neonatal rats were used to prepare primary cardiomyocytes; the procedure was performed as described previously.^28^ The Wistar rats used in the experiments were provided by the Experimental Animal Holding of Jilin University and fed by the Animal Laboratory Center at the Daqing Campus, Harbin Medical University.^29^ All experiments involving animals should be conducted in accordance with *the regulations of the ethics committee of Harbin Medical University*.

### 2.3 Expression and characterization of rTβ_4_ and PEG‐rTβ_4_

#### 2.3.1 Expression and characterization of rTβ_4_

The gene encoding rTβ_4_ was synthesized using genetic engineering methods (Suzhou Hongxun Biotechnology Co., Ltd., Suzhou, China). To build two tandem repeats of Tβ_4_, the Histidine(H)‐Lysine(K) Carbonyldiimidazole (HKCDI) amino acid sequence and the glycine‐serine linker gene were combined with the rTβ_4_ sequence, as shown in Figure 1a, and cloned into the pET‐28a (+) vector. The recombinant plasmid was then transinfected into *E. coli* BL21 (DE3), induced with Isopropyl‐β‐D‐1‐thiogalactopyranoside (0.25 mM), and ÄKTATM prime plus (GE Healthcare, USA) equipped with a Ni‐sepharose column was used to purify rTβ_4_. Finally, rTβ_4_ was identified by western blot and mass spectrometry (matrix‐assisted laser desorption‐ionization time of flight mass spectrometry [MALDI‐TOF MS], Shanghai Applied Protein Technology Co., Ltd., China).

#### 2.3.2 A site‐specific PEGylation of rTβ_4_

PEG was activated to prepare N‐terminal PEG‐rTβ_4_. Briefly, PEG‐MAL (Xi'An Ruixi Biological Technology Co., Ltd., Xi'an, China) was dissolved in phosphate‐buffered saline (PBS) solution (4 mg/mL, pH 8.0), comprising 0.01% Tween 80. PEG‐MAL in this solution was reacted with rTβ_4_ in the PBS solution (4 mg/mL, pH 8.0). The reaction system included 0.05 μmol/mL TCEP as thiol protectant and catalyst, and was stirred overnight under a nitrogen atmosphere at 8°C. After the reaction, the product was diluted with PBS containing 0.01% Tween 80 (pH 8.0) at a 4:1 ratio (product to PBS). The diluted product was purified by size‐exclusion chromatography using an ÄKTATM prime plus system equipped with a Sephadex G25 column. Subsequently, the purified product was concentrated three times by ultrafiltration using a Merck Millipore filter with a 10 kDa molecular weight cut‐off at 5000 g for 15 min each time. The molecular weight of the purified product was identified by MALDI‐TOF MS.

#### 2.3.3 Sodium dodecyl sulfate polyacrylamide gel electrophoresis analysis of PEG‐rTβ_4_

The resulting rTβ_4_ and PEG‐rTβ_4_ were analyzed with sodium dodecyl sulfate polyacrylamide gel electrophoresis (SDS‐PAGE, 15% polyacrylamide gel). Then, the gel was sequentially stained with Coomassie Brilliant Blue R‐250 and a 3% iodine‐potassium iodide (I‐KI) solution. The purity and PEGylation proportion of the sample were assessed from Coomassie‐ and iodine‐staining images. The amount of rTβ_4_ in the purified products was analyzed by gray scanning of the Gel Imaging System (AI600‐Amersham, General Electric, USA).

#### 2.3.4 Analysis with thermogravimetric analyzer

A 5 mg sample of a physical mixture of PEG‐MAL and rTβ_4_ (containing 0.85 mg PEG‐MAL and 4.15 mg rTβ_4_) was prepared for analysis. The analysis condition was as follows: the heating rate was 10°C/min; the flow rate of N_2_ was about 0.33 mL/s; and the termination temperature was 600°C. Weigh every particular sample, and then the thermal gravimetric analysis (TGA) and differential scanning calorimetry (DSC) curves were recorded to acquire experimental results. Each sample was repeated three times using by Thermal Gravimetric Analyzer (TGA2/SDTA851, METTLER‐TOLEDO, Switzerland).

### 2.4 Cytological experiments

#### 2.4.1 Cell proliferation assay

H9c2 cells (derived from embryonic rat heart tissue) were incubated in 96‐well plates. The number of cells was approximately 1 × 10^3^ per well. H9c2 cells were incubated with 0, 1, 5, 10, and 20 μg/mL of rTβ_4_ without serum at 37°C in 5% CO_2_, and then were stained with Hochest 33,258 for 60 s at room temperature (the final volume of 0.001%). The numbers of H9c2 cells in the groups were determined using the Cytation 5 Cell Imaging Multi‐Mode Reader (Cytation 5, BioTek, USA) at the given time points.

#### 2.4.2 Cell scratch test

In the cell scratch test, H9c2 cells were incubated in 12‐well plates at approximately 5 × 10^4^ cells per well and cultured for 24 h. After incubation, a pipette tip was used to make a scratch in each well. H9c2 cells were incubated in 0, 1, 2, and 5 μg/mL of rTβ_4_ and 2.4 μg/mL of PEG‐rTβ_4_ (equal to rTβ_4_ of 2 μg/mL) at 37°C in a 5% CO_2_ atmosphere for 24 h. Images of wound closure were taken at 6, 12, 18, and 24 h after the scratch using a microscope (Leica M60FL, Germany). The migration area of H9c2 cells was analyzed using ImageJ. The migration area was calculated by subtracting the 0‐h area from the subsequent time‐point areas. This calculation used data from three replicates, which were analyzed with GraphPad Prism 8.0 (*n* = 3).

#### 2.4.3 Apoptosis detection of cardiomyocytes

H9c2 cells were seeded in 12‐well plates at a density of approximately 5 × 10^4^ cells per well, while primary cardiomyocytes were also seeded in 12‐well plates at a density of 1 × 10^4^ cells per well. After 48 h of incubation, hypoxic preconditioning to induce cell apoptosis was performed in a tri‐gas incubator with an oxygen concentration set at 2% for 24 h. The normoxic control group was cultured under standard conditions. Concurrently, cells were incubated separately with 2.4 μg/mL of PEG‐rTβ_4_, 2.0 μg/mL of rTβ_4_, or 0.4 μg/mL of PEG‐MAL (the control group under hypoxia was free, while the positive control was incubated with carbonyl cyanide 3‐chlorophenylhydrazone). Each group was repeatedly tested three times. After experiments, flow cytometry analysis (AccuriC6, BD, USA) of H9c2 cells and fluorescence imaging (Leica M60FL, Germany) of primary cardiomyocytes were performed using a JC‐1 kit (Peking Beyotime Biotechnology Co., Ltd., Peking, China) to assess mitochondrial membrane potential.

### 2.5 Animal experiments

#### 2.5.1 Establishment of myocardial infarction model

To prepare the MI animal model, first, a 10% chloral hydrate (350 mg/kg) was intraperitoneally injected to induce narcosis. Then, the left anterior‐descending artery (LAD) of male adult Wistar rats (body weight 200 g) was ligated, while the sham‐operated animals underwent thoracotomy without LAD ligation. At 48 h after MI model establishment, MI induction was confirmed by echocardiography. The ejection fraction (EF) of the operated rats ranged from 30% to 60%, indicating the successful establishment of MI models.^30^ After MI, the infarction area discolored after reacting with triphenyl tetrazolium chloride (TTC), and the ST segment of the electrocardiograph (ECG) was elevated. Two percent TTC staining of the ventricular tissue section and ECG recording using BL‐410 biological function experiment systems were performed (Figure S3A,B).^29^

#### 2.5.2 Experimental grouping and administration

To assess the therapeutic effect of PEG‐rTβ_4_, the MI model rats were established for the subsequent experiments. Rats were randomly divided into the following groups: MI + PBS group (*n* = 6), MI + rTβ_4_ group (*n* = 6), MI + PEG‐rTβ_4_ group (*n* = 6), MI + PEG‐rTβ_4_ + LY group (*n* = 6), and sham‐operation group (*n* = 6); rTβ4 at 2 mg/kg and PEG‐rTβ_4_ at 2.4 mg/kg (contained 2 mg/kg of rTβ_4_) was injected via tail vein every 3 days, and LY (Shanghai Duma Biotechnology Co., Ltd., Shanghai, China) at 2 mg/kg was administered by IV bolus injection once a day.

#### 2.5.3 Echocardiography evaluation

Before administering the drug, transthoracic echocardiography was performed at preset time points (Day 2, 5, 9, 16, 23, and 30). We obtained two‐dimensional (2D) and M‐mode echocardiographic images using a linear transducer (15‐MHz, VisualSonics Vevo 2100, Toronto, ON, Canada). Cardiac dimensions, two parameters of the left ventricular (LV) end‐systolic diameter (LVESD) and the left ventricular end‐diastolic diameter (LVEDD), were assessed in the short‐axis view at the papillary muscle level using M‐mode tracing. Subsequently, left ventricular end‐systolic volume (LVESV) and left ventricular end‐diastolic volume (LVEDV) were estimated using these measurements. All assessments were performed in a blinded manner, and results were averaged over five consecutive cardiac cycles. Generally, the Teichholz formula is commonly used to assess LV volume: *V* = (7 × *D* ^3^)/(2.4 + *D*), where *D* is the LV short‐axis diameter and *V* is the volume. Cardiac contractile function was assessed by calculating the left ventricular ejection fraction (LVEF) parameter using computer algorithms. LVEF was calculated using the formula: LVEF = [(LVEDV − LVESV)/LVEDV] × 100%.

### 2.6 Histological analysis

#### 2.6.1 Preparation of frozen sections

After continuous administration to MI model rats for 4 weeks, the rats were euthanized, and the hearts, brains, livers, lungs, spleens, and kidneys of the rats were removed. The hearts were dissected to isolate the tissue below the ligation site, which was then sectioned separately. All organs were dehydrated for 48 h in 30% sucrose solution, fixed for 24 h with 4% paraformaldehyde, and then embedded in embedding media. Subsequently, 5 μm‐thick transverse cryosections were prepared and mounted on glass slides. These frozen sections were used for histological and immunofluorescence staining.

#### 2.6.2 Hematoxylin/eosin staining and Masson's trichrome staining

Frozen sections of organs were sequentially hydrated with 100%, 90%, 80%, and 70% ethanol. The sections, selected for routine testing of each organ, were stained with hematoxylin–eosin (HE). In the final step, xylene was used to clear the sections, which were then mounted and coverslipped using Rhamsan mounting medium. The Masson's Trichrome Staining Kit (Peking Solarbio Biotechnology, Peking, China) was used to assess cardiac interstitial collagen deposition for fibrosis determination in the infarct border zone, following the specific experimental procedure described in the manufacture's instructions.

#### 2.6.3 Immunohistochemical and immunofluorescent assay

An antibody against VEGF (Peking Bioss Biotech, Co., Ltd., Peking, China) was used to quantify the VEGF level. In contrast, the antibodies against CD31 (Wuhan Huamei Bioengineering Co., Ltd., Wuhan, China) and α‐SMA (Peking Bioss Biotech, Co., Ltd., Peking, China) were used to evaluate capillary and arteriolar density, respectively. Additionally, 4′,6‐diamidino‐2‐phenylindole (DAPI) was used to stain nuclei, and DiO was used to stain cell membranes. The VEGF level and the densities of blood vessels in the ischemic penumbra were assessed by immunohistochemical and immunofluorescent assays, respectively. Immunofluorescence images were obtained from a live‐cell workstation (Leica AF6000‐AF70, Germany); the fibrosis area, VEGF level, and vessel density were quantified using ImageJ.

### 2.7 Western blot analysis

Heart tissue of rats was lysed in radio‐immunoprecipitation assay buffer supplemented with 1 mmol Phenylmethylsulfonyl fluoride as a protease inhibitor and 1 mmol phosphatase inhibitor to extract total proteins. Protein concentrations were determined using the bicinchoninic acid (BCA) protein assay protocol, and proteins were separated by SDS‐PAGE. Proteins separated within the gel were then transferred to a polyvinylidene fluoride (PVDF) membrane, which was activated by methanol for 30 s prior to use. After blocking with 5% skim milk powder, the membranes were incubated overnight at 4°C with primary antibodies, including anti‐β‐actin, anti‐Akt, and anti‐p‐Akt (mixed and incubated simultaneously); anti‐Bax and anti‐Bcl‐2 (mixed and incubated simultaneously); and anti‐cleaved caspase‐3 and anti‐cleaved caspase‐9 antibodies (Abcam's Heilongjiang Regional agent, China). Subsequently, membranes were incubated with the appropriate secondary antibodies for 2 h at room temperature. The blots were developed using a chemiluminescent substrate and visualized with a gel imaging system.^31^

### 2.8 Long circulation of PEG‐rTβ_4_

MI model rats were injected with PEG‐rTβ_4_ and rTβ_4_ via the tail vein at a dose of 2 mg/kg based on rTβ_4_. Whole blood samples were collected at different time points. Serum was separated by centrifugation and then concentrated using an ultrafiltration centrifuge tube. The concentrations of PEG‐rTβ_4_ and rTβ_4_ in the serum were assessed by Western blot. A histidine‐tagged antibody was used for the semiquantitative analysis of PEG‐rTβ_4_ and rTβ_4_.

### 2.9 Statistical analysis

All statistical analyses were performed using GraphPad Prism 8 (La Jolla, CA). All data were exhibited as the mean ± standard error of the mean. One‐way analysis of variance was conducted across all investigated groups before post hoc tests with Bonferroni correction, and all two‐group comparisons were made. *p* < 0.05, 0.01, and 0.001 were considered statistically significant, highly significant, and extremely significant, respectively.

## 3 RESULTS AND DISCUSSION

Ischemia and hypoxia are the leading causes of myocardial tissue damage. Reversing the two factors is a current of developing cardiovascular therapeutics. Biological and polymer‐modified biological drugs have contributed significantly to the treatment of myocardial ischemic diseases.^32^ Furthermore, biological polymer‐modified prodrugs have become a new force in the post‐development era of biological drugs, gradually replacing unmodified biological drugs.

PEG is the leading polymer for biological drug modification, and site‐specific PEGylation is the most effective method to ensure the final desired protein is of high purity. Recombinant Tβ_4_ is the earliest application of the thymosin β family in clinical practice, and it has been used in several research studies across various fields, including inhibiting scar formation, myocardial injury repair, hair follicle regeneration, skin wound healing, neurogenesis, and corneal repair^33^, ^34^, ^35^, ^36^, ^37^, ^38^, ^39^ Due to the extensive biological effects and high exploitation potential of Tβ4, we designed a new recombinant Tβ4, rTβ_4_.

### 3.1 Preparation and characterization of rTβ_4_ and PEG‐rTβ_4_

**FIGURE 2:**
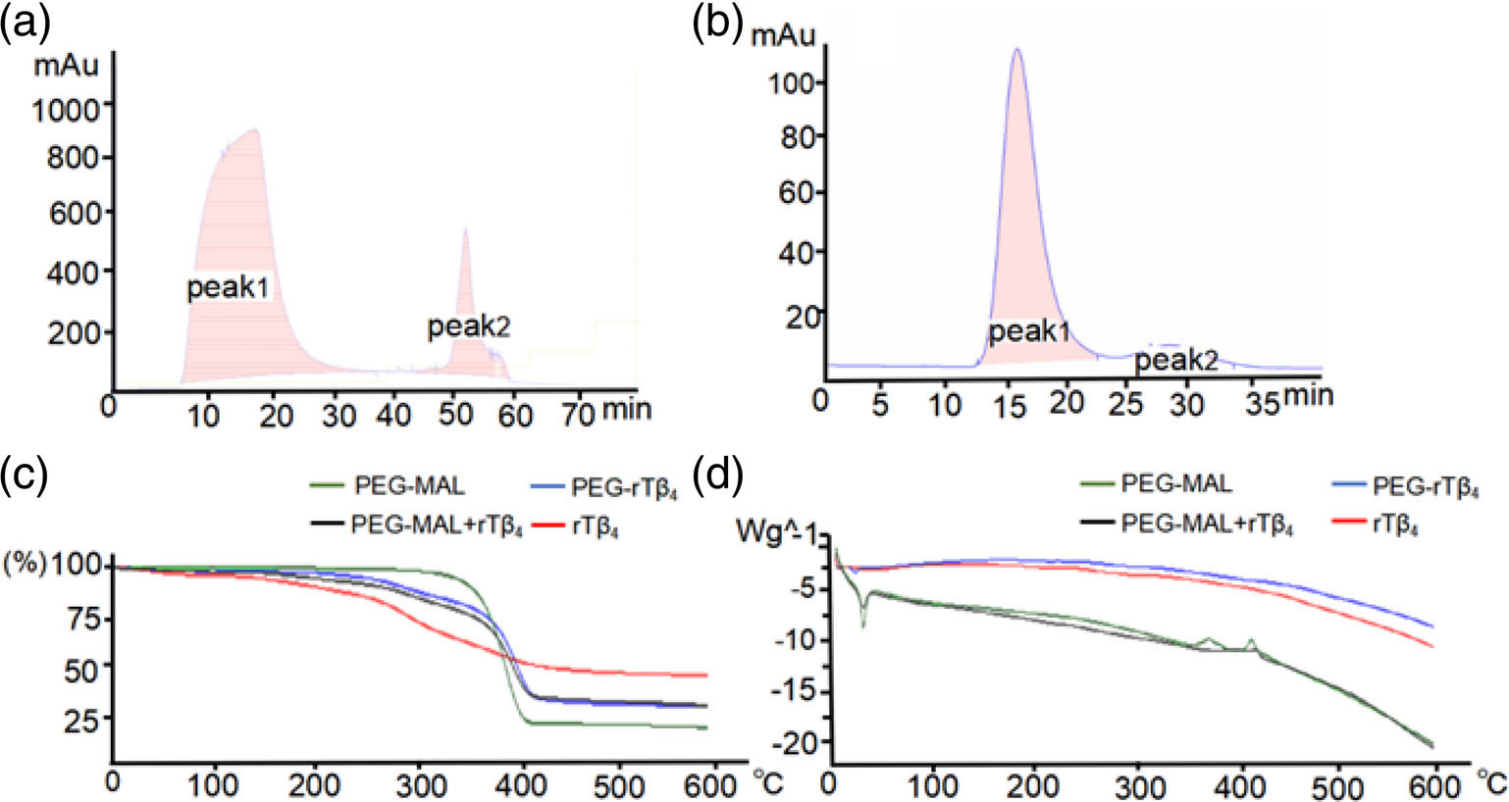
Purification and thermodynamics properties of recombination thymosin β_4_ (rTβ_4_) and PEGylated recombination thymosin β‐4 (PEG‐rTβ_4_) (*n* = 3). (a) The chromatogram of the miscellaneous protein peak (peak 1) and purified rTβ_4_ (peak 2). (b) The chromatogram of purified PEG‐rTβ_4_ (peak 1) and rTβ_4_ (peak 2). (c) Thermal gravimetric analysis curves for polyethylene glycol 2000 with maleinimide (PEG‐MAL) (green line), rTβ_4_ (red line), the physical mixture of PEG‐MAL and rTβ_4_ (black line), and PEG‐rTβ_4_ (blue line). (d) Differential scanning calorimetry curves for PEG‐MAL (green line), rTβ_4_ (red line), the physical mixture of PEG‐MAL and rTβ_4_ (black line), and PEG‐rTβ_4_ (blue line).

The HKCDI gene sequence, inserted into the Tβ4 gene, was used to increase rTβ4 expression and to introduce a Cys residue as the specific modification site. The thiol group of a single Cys residue in the rTβ_4_ peptide chain was the sole designed position that conjugated with PEG‐MAL (mPEG‐MAL, MW, 2000 Da) to produce PEG‐rTβ_4_. In addition, a hexahistidine tag was added to the carboxyl terminus of the amino acid sequence to facilitate purification, identification, and quantification of rTβ4 in serum using the histidine antibody. The schematic structure of PEG‐rTβ_4_ is shown in Figure 1. We have further successfully prepared rTβ_4_ using prokaryotic expression systems; the chromatogram of rTβ_4_ purified was shown in Figure 2a (peak 2), and rTβ_4_ was first identified by western blot (Figure S1A). The percentage of rTβ_4_ to the total proteins (rTβ_4_ peak area/[rTβ_4_ peak area + miscellaneous protein peak area]) was about 13.9%. Figure 2b was a chromatogram of the purified PEG‐rTβ_4_ (peak 1). In Figure 2b, PEG‐rTβ_4_ has a single peak with a more significant proportion of peak area (rTβ_4_: PEG‐rTβ_4_, 5.4%:94.6%). The molecular weights were confirmed by MALDI‐TOF MS (Fig. S2A,B). The experimental molecular weights of the two peptides (rTβ_4_, MW = 11.74 kDa; PEG‐rTβ_4_, MW = 13.86 kDa) matched well with the theoretical values predicted by protpi.ch/Calculator/ProteinTool. Furthermore, on staining gels, both purified rTβ_4_ and purified PEG‐rTβ_4_ were single strips. The purity of PEG‐rTβ_4_ was above 90%, as determined by gel scan analysis in Figure S1B, proving that PEG was site‐specifically attached to rTβ_4_ via the single Cys residue in the entire amino acid sequence. The preparation processes, including PEG modification and secondary purification, were carried out at low temperatures to ensure the sample's purity and activity for the subsequent tests.

TGA and DSC were utilized to measure changes in thermodynamic parameters of rTβ_4_ before and after PEG modification. In the TGA curve (Figure 2c), the rTβ_4_ sample began to lose weight at 150°C, while PEG‐rTβ_4_ at 200°C, suggesting that the PEGylation increases the thermal stability of rTβ_4_. When the temperature exceeded 430°C, the TGA curves of PEG‐rTβ_4_ and the physical mixture of mPEG_2000_‐MAL (PEG) + rTβ_4_ (1:1) coincided; they had the same mass loss ratio, indicating that the PEGylation proportion is 1:1 and no non‐specific modification in PEG‐rTβ_4_ occurred. In the TGA curves, PEGylation increased the thermal stability of PEG‐rTβ_4_, suggesting that its stability in vitro and in vivo could be improved. Further, the PEG‐rTβ4's in vitro validity period may be extended, and its curative effect may be increased in vivo. On the other hand, good modification effects and product purity were also confirmed by the TGA curves.

In Figure 2d, the DSC curves of PEG‐rTβ_4_ and rTβ_4_ showed a similar trend, with no apparent phase transition observed. These results suggest that a site‐specifically PEG‐rTβ_4_ via the thiol group with small‐molecular‐weight PEG does not significantly alter the crystal and spatial structures of rTβ_4_. After analyzing the DSC curves, we concluded that the thiol‐site‐specifically PEGylated would be unable to alter the crystal and spatial structures of rTβ_4_. Thus, the modification had little effect on the interaction between PEG‐rTβ4 and actin, thereby maintaining PEG‐rTβ4's efficacy.

### 3.2 PEG‐rTβ_4_ promotes H9c2 proliferation and migration

**FIGURE 3:**
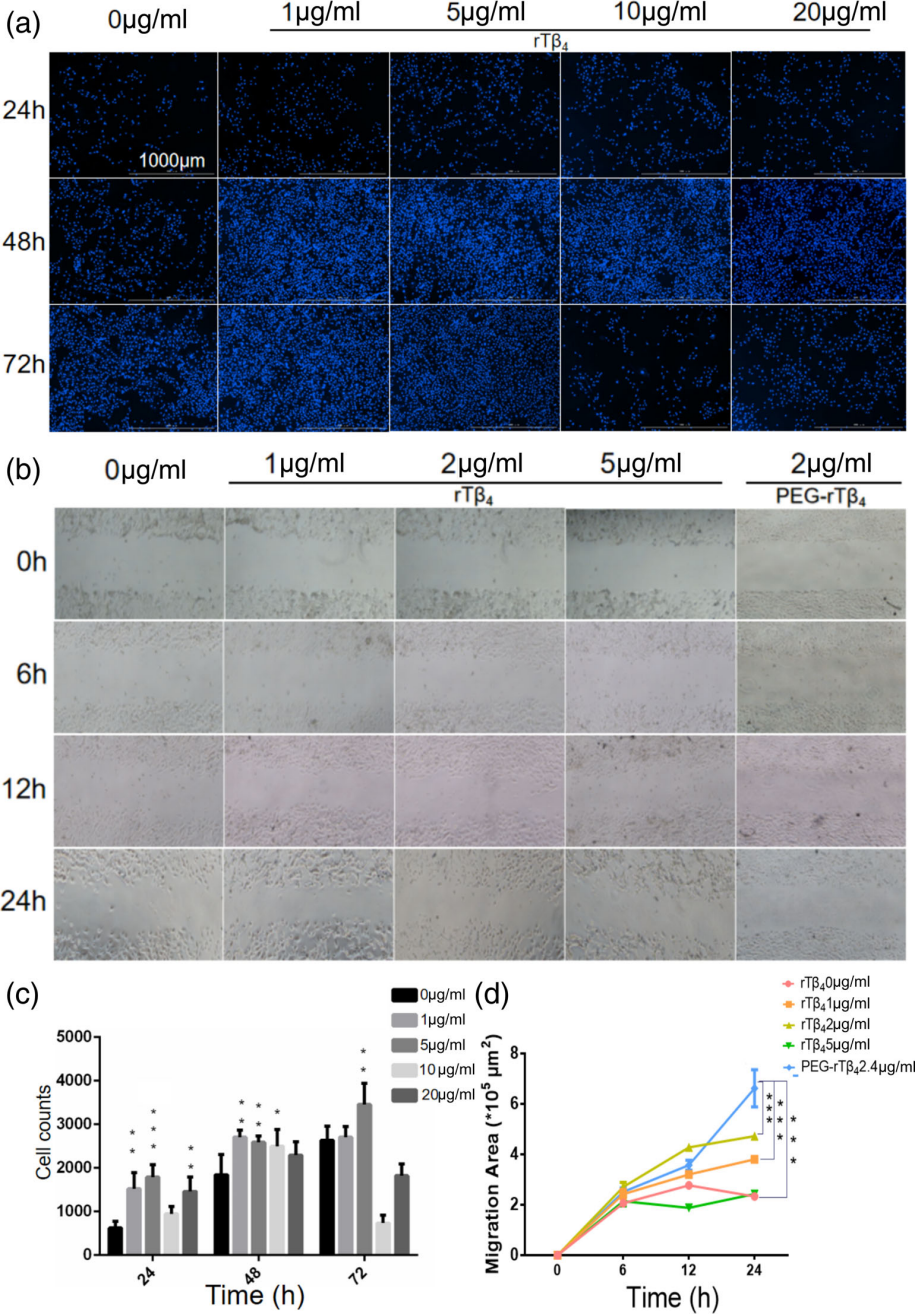
Recombination thymosin β‐4 (rTβ_4_) promotes the proliferation and migration of H9c2 cells (a myocardial cell line) at different concentrations (*n* = 3). (a) Images showing cellular proliferation at doses of 1, 5, 10, and 20 μg/mL of rTβ_4_. (b) Images showing the migration of H9c2 cells at 0, 6, 12, and 24 h after incubation with rTβ_4_ and PEGylated rTβ_4_ (PEG‐rTβ_4_). (c) Statistical analysis of (a): At 24 h, **p < 0.01 for 0 μg/mL (control) versus 1 and 20 μg/mL, and ***p < 0.001 for 0 versus 5 μg/mL; at 48 h, *p < 0.01 for 0 versus 10 μg/mL, and **p < 0.01 for 0 μg/mL versus 1 and 5 μg/mL; at 72 h, **p < 0.01 for 0 μg/mL versus 5 μg/mL. (d) Statistical analysis of (b) shows that at 24 h, treatment with 2.4 μg of PEG‐rTβ_4_ significantly differs from all other doses of PEG‐rTβ_4_ groups (***p < 0.001).

The H9c2 cells were incubated with different amounts of rTβ_4_. Cell proliferation assays showed that rTβ_4_ at concentrations of 1–5 μg/mL significantly enhanced cell proliferation at 24, 48, and 72 h after incubation. However, the effects became less pronounced at higher concentrations (10 or 20 μg/mL, Figure 3a,b). Tβ_4_ exhibits a significant increase in toxicity with increasing concentration, as reported in previous studies.^40^ The cell counting data at 24 h after incubation also supported the occurrence of overdose‐related cytotoxic effects, especially in the cells treated with 10 and 20 μg/mL of rTβ_4_. As incubation time increased, the number of cells treated with 10 and 20 μg/mL of rTβ_4_ significantly decreased. All results indicate that cell death was caused by high concentrations of rTβ_4_ (10 or 20 μg/mL, Figure 3a,b), rather than by nutrient depletion. Therefore, we chose concentrations of 1–5 μg/mL for scratch experiments. We found that both rTβ_4_ (1–5 μg/mL) and PEG‐rTβ_4_ (2.4 μg/mL) significantly improved cell migration within 12 h (Figure 3c). The effect of PEG‐rTβ_4_ became clearer after 24 h (Figure 3d). Thus, we selected 2.4 μg/mL of PEG‐rTβ_4_ and 2.0 μg/mL of rTβ_4_ for further experiments, noting that 2 μg/mL of rTβ_4_ equals 2.4 μg/mL of PEG‐rTβ_4_ in molar concentration (Figure S2).

### 3.3 PEG‐rTβ_4_ inhibits hypoxia‐induced apoptosis of cardiomyocytes

**FIGURE 4:**
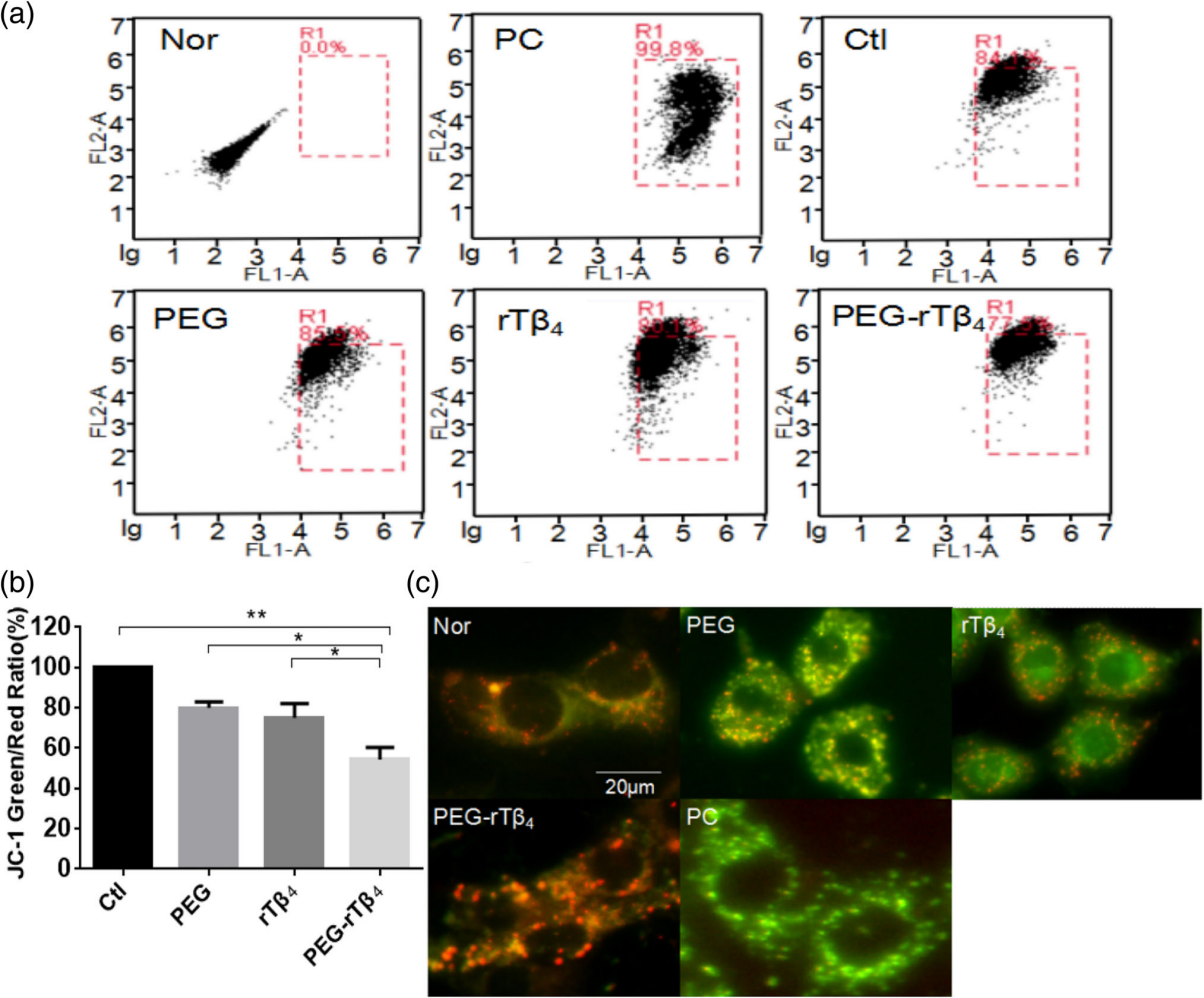
PEGylated recombination thymosin β‐4 (PEG‐rTβ_4_) suppresses apoptosis in H9c2 cells and primary cardiomyocytes (*n* = 3). (a) Apoptosis in H9c2 cells was analyzed by flow cytometry. H9c2 cells were incubated with carbonyl cyanide, *m*‐chlorophenyl hydrazone (positive control, PC), polyethylene glycol (PEG), rTβ_4_, and PEG‐rTβ_4_, respectively. Nor represents normal cells without treatment, while Ctl represents hypoxia‐induced H9c2 cells. (b) Statistical analysis of (a). The ratio of apoptotic cells (green) to average total cells (red) was normalized to the Ctl data. *p < 0.05, PEG‐rTβ_4_ versus PEG or rTβ_4_, **p < 0.01, PEG‐rTβ_4_ versus Ctl. (c) Fluorescence imaging of apoptotic primary myocardial cells. The mitochondria of apoptotic cells stained by JC‐1 are green, while those of non‐apoptotic cells are red. PC indicates cells treated with carbonyl cyanide, *m*‐chlorophenyl hydrazone (positive control), while Nor represents normal cells without treatment.

We found that rTβ_4_ or PEG‐rTβ_4_ could promote cell proliferation and migration. On this basis, we further demonstrated the anti‐apoptotic effects of PEG‐rTβ4 using H9c2 and primary myocardial cells, confirming that PEG‐rTβ4 can maintain normal cardiac function after MI. Apoptosis of H9c2 was shown in Figure 4a. In the control (Ctl), PEG, rTβ_4_, and PEG‐rTβ4 groups, their early apoptosis ratio was 84.1%, 85.6%, 80.1%, and 77.5%, respectively, suggesting that rTβ_4_ and PEG‐rTβ_4_ can inhibit hypoxia‐induced early apoptosis. The ratio of early apoptotic cells (green staining) to total cells (red staining) was calculated based on the flow cytometry data in Figure 4a. All ratios were then normalized to the control, as shown in Figure 4b. The results showed that PEG‐rTβ4 was significantly more effective than rTβ_4_. Primary cardiomyocytes were used to observe hypoxia‐induced apoptosis by fluorescence microscopy (Figure 4c); mitochondria of apoptotic cells were stained with JC‐1, a green fluorescent probe, while those of non‐apoptotic cells were stained red. Generally, the mitochondrial membrane stained with JC‐1 exhibited a color change from red, through orange and yellow, to green, indicating a decrease in mitochondrial membrane potential caused by hypoxia. As shown in Figure 4c, the mitochondrial membrane in cells treated with rTβ_4_ displayed a more intense red color than that in the PEG group. Fluorescent imaging provides limited information from the samples tested. To supplement these observations, flow cytometry provided quantitative data on the extent of apoptosis inhibited by rTβ_4_ and PEG‐rTβ_4_. Together, the flow cytometry and fluorescence imaging results indicate that PEG‐rTβ_4_ can significantly inhibit hypoxia‐caused myocardial apoptosis.

### 3.4 PEG‐rTβ_4_ preserves cardiac function after MI

**FIGURE 5:**
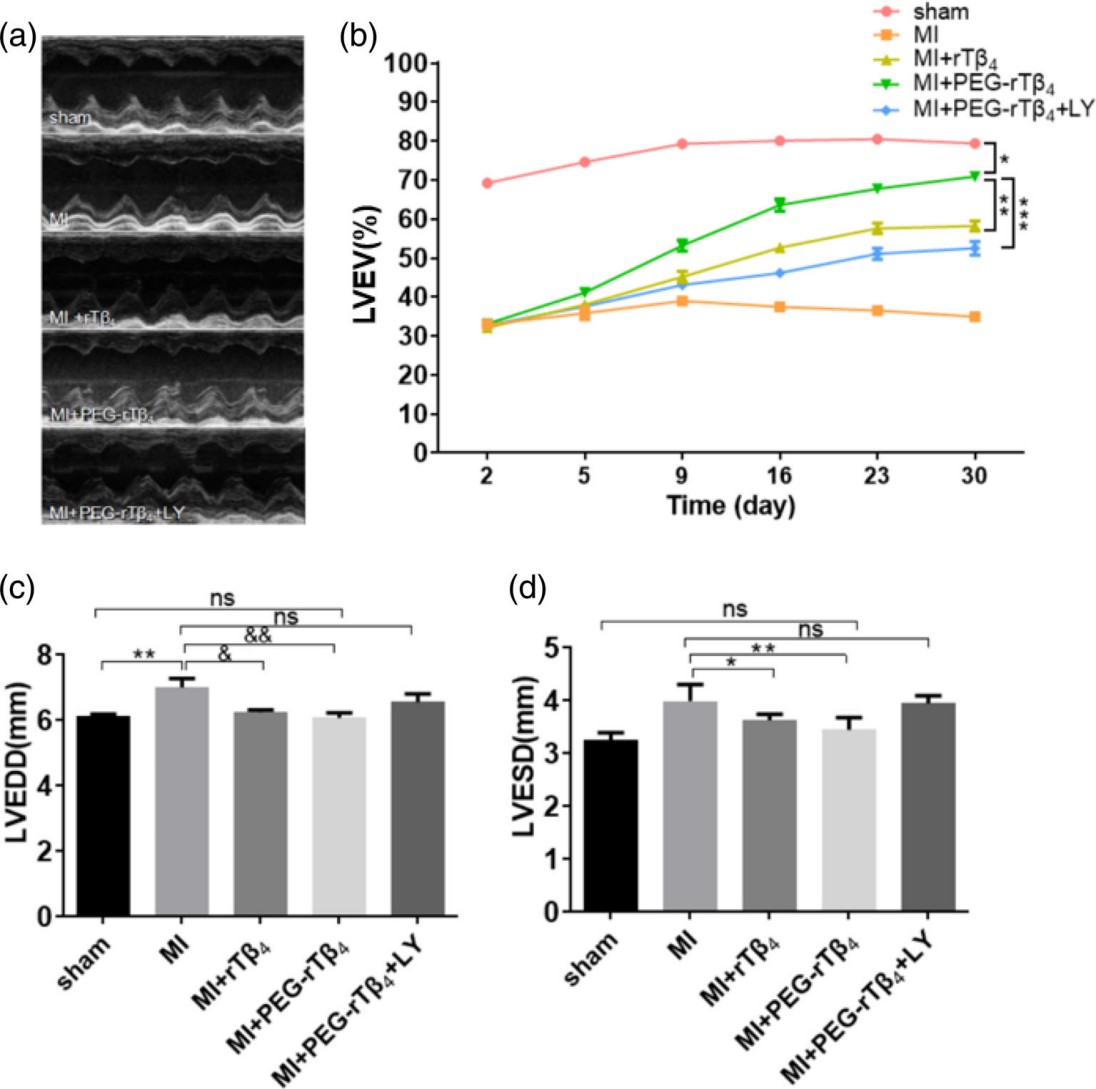
PEGylated recombination thymosin β‐4 (PEG‐rTβ_4_) restores the cardiac left ventricular structure and function in myocardial infarction (MI) rats (*n* = 6). (a) Echocardiograph of animals after continuous treatment for 4 weeks. (b) Statistical analysis of left ventricular ejection fraction (LVEF). *p < 0.05, sham versus MI + PEG‐rTβ_4_ at 30 days post‐administration; **p < 0.01, MI + rTβ_4_ versus MI + PEG‐rTβ_4_; ***p < 0.001, MI + PEG‐rTβ_4_ versus MI + PEG‐rTβ_4_ + LY at 30 days. (c) Statistical analysis of left ventricular end‐diastolic dimensions (LVEDD). No significant differences (ns) were observed between sham versus MI + PEG‐rTβ_4_ and MI versus MI + PEG‐rTβ_4_ + LY groups; **p < 0.01, sham versus MI; ^&^ *p* < 0.05, MI versus MI + rTβ_4_; ^&&^ *p* < 0.01, MI versus MI + PEG‐rTβ_4_ rat 30 days. (d) Statistical analysis of left ventricular end‐systolic dimensions (LVESD). No significant differences (ns) were observed between sham versus MI + PEG‐rTβ_4_ and MI versus MI + PEG‐rTβ_4_ + LY groups; *p < 0.05, MI versus MI + rTβ_4_; **p < 0.01, MI versus MI + PEG‐rTβ_4_ rats 30 days.

After MI, cardiac function and structure parameters in the MI model rats were assessed by echocardiography. As shown in M‐mode echocardiography (Figure 5a), after being treated with MI + rTβ_4_, MI + PEG‐rTβ_4_ + LY, and MI + PEG‐rTβ_4_, respectively, every 3 days for 4 weeks, the thickness of the LV free wall failed to exert significant improvement in the MI + rTβ4 and MI + PEG‐rTβ4 + LY groups compared to the MI group. Nevertheless, treatment with PEG‐rTβ4 could statistically improve cardiac function, as evidenced by higher LVEF values (Figure 5b) compared with MI, MI + rTβ4, or MI + PEG‐rTβ4 + LY groups. LVEF, an essential index of cardiac function, increased significantly after 2 weeks of PEG‐rTβ4 treatment and returned to near‐normal levels after 3 weeks of treatment. Additionally, diastolic and systolic heart volumes were evaluated using the structural parameters LVEDD and LVESD at 30 days (Figure 5c,d). After MI, LVEDD and LVESD of animals treated with MI + PEG‐rTβ_4_ were similar to those of the sham groups. These changes indicate an association with significant improvement in cardiac function in MI rats. However, the effects may be attenuated by LY, an Akt inhibitor. The preservation of cardiac function of PEG‐rTβ_4_ was more significant than that in the rTβ_4_ or PEG‐rTβ_4_ + LY inhibitor group.

The data showed that treatment with rTβ_4_ every 3 days cannot sustain significant cardioprotective effects in vivo. These consequences also highlight the value of PEG modification and PEG‐rTβ_4_ as a long‐circulating protein therapeutic. The activation of Akt was antagonized by LY, as confirmed in vitro. However, the in vivo effect of LY remains unknown. When PEG‐rTβ_4_ and LY were administered simultaneously, LY inhibited PEG‐rTβ_4_‐induced Akt activation and induced the expression of pro‐apoptotic proteins (Figure 8), thus inhibiting cell proliferation (Figure 3) and promoting cell apoptosis (Figure 4), which slacked the effect of PEG‐rTβ_4_. The in vivo action of rTβ4 is less evident than in vitro cell experiments, which may be due to degradation of the complex enzyme system and the complement system in the animal body. However, the effects of PEG‐rTβ_4_ were much longer than those of rTβ_4_ in preserving the cardiac function of MI rats.

### 3.5 PEG‐rTβ_4_ alleviates cardiac remodeling and enhances myocardial angiogenesis

**FIGURE 6:**
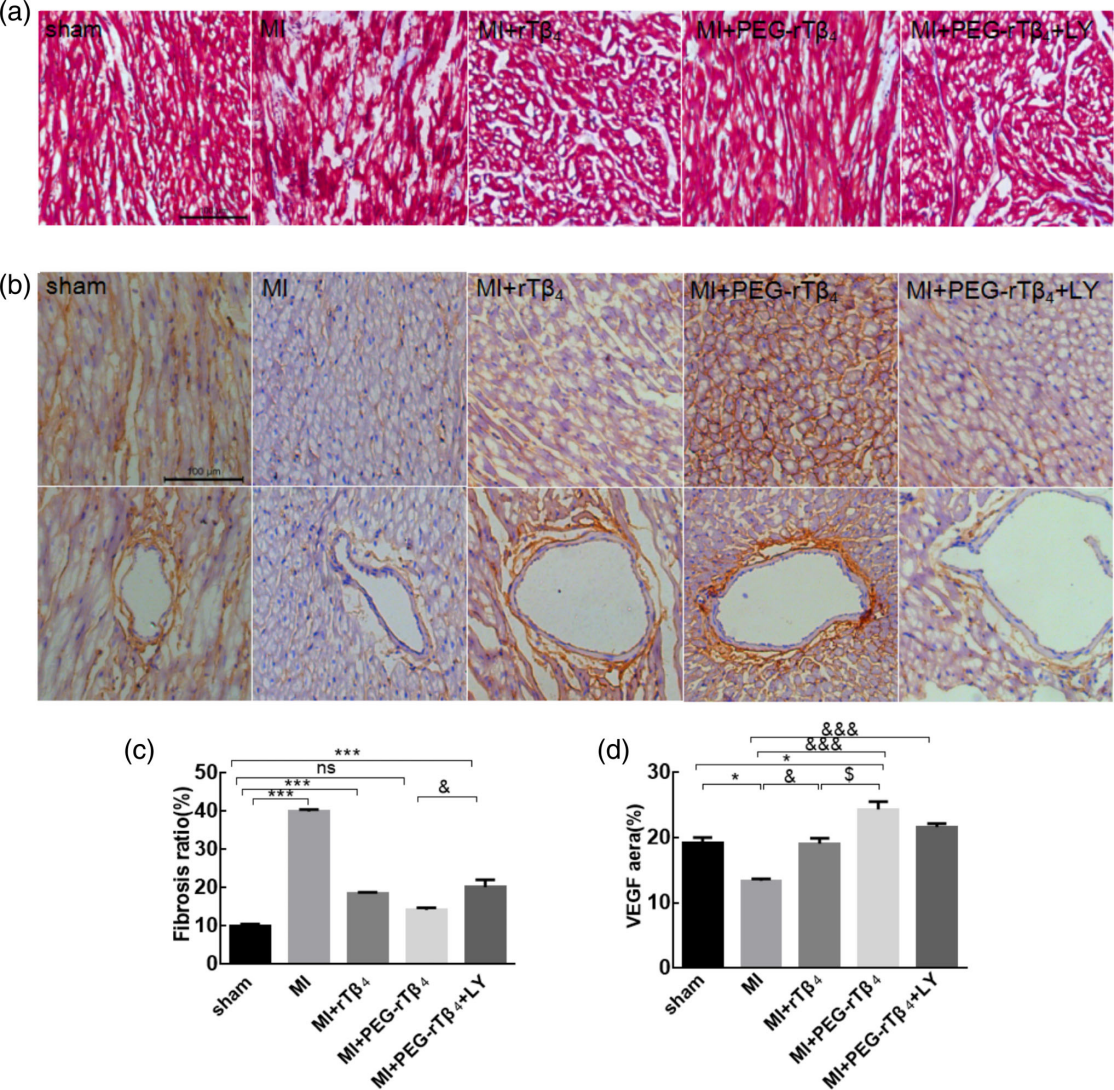
PEGylated recombination thymosin β‐4 (PEG‐rTβ_4_) prevents left ventricular (LV) remodeling and stimulates the production of vascular endothelial growth factor (VEGF) (*n* = 3). (a) Masson's trichrome staining of cardiac LV from myocardial infarction (MI) rats after the surgery and treatment with rTβ_4_, PEG‐rTβ_4_, and PEG‐rTβ_4_ + LY, respectively. (b) VEGF expression was detected by immunohistochemical staining of cardiac LV from MI rats after the surgery and treatment with rTβ_4_, PEG‐rTβ_4_, and PEG‐rTβ_4_ + LY, respectively. The upper lane shows heart tissue staining, and the lower lane shows blood vessels in the heart. (c) Quantification of fibrosis ratio from (a). ***p < 0.001, sham versus MI, MI + rTβ_4_, or MI + PEG‐rTβ_4_ + LY; no significance (ns), sham versus MI + PEG‐rTβ_4_; ^&^ *p* < 0.05, MI + PEG‐rTβ_4_ versus MI + PEG‐rTβ_4_ + LY. (d) Statistical analysis of (b) (tissue data only, not blood vessels): *p < 0.05, sham versus MI or MI + PEG‐rTβ_4_; ^&^ *p* < 0.05, MI versus MI + rTβ_4_; ^&&&^ *p* < 0.001, MI versus MI + PEG‐rTβ_4_ or MI + PEG‐rTβ_4_ + LY; ^$^ *p* < 0.05, MI + rTβ_4_ versus MI + PEG‐rTβ_4_.

Cardiac remodeling was evaluated by fibrosis ratio (% area); as time passes, the affected myocardial tissue is gradually replaced by collagen fibers, which also explains cardiac remodeling after MI. Masson's trichrome staining examined sections of myocardial tissue from each group in vivo. The results showed that PEG‐rTβ_4_ lowered the fibrosis ratio in the ischemic penumbra of MI. However, the inhibition of fibrosis by PEG‐rTβ_4_ + LY and rTβ_4_ was weaker (Figure 6a,c). Moreover, the rationality of LVEDD and LVESD dimensions was an essential feature for alleviating ventricular remodeling, as evidenced by reduced LV dimensions and cardiac size (Figure 5c,d).

Angiogenesis is a critical factor in cardiac functional compensation post‐MI; VEGF can trigger the growth of new capillaries under low‐oxygen conditions, which is directly related to the rate of angiogenesis. Ex vivo immunohistochemical staining was performed to assess the VEGF level. In the ischemic penumbra of MI, the expression of VEGF was markedly increased in the MI + PEG‐rTβ_4_ group compared with other groups in cardiac intercellular spaces; rTβ_4_ can also increase the VEGF level in the heart tissue; whereas after being treated with LY, this effect of PEG‐rTβ_4_ has been discounted (Figure 6b,d). We were pleasantly surprised that this phenomenon was even more pronounced around the blood vessels in the heart tissue (Figure 6b, down the lane).

**FIGURE 7:**
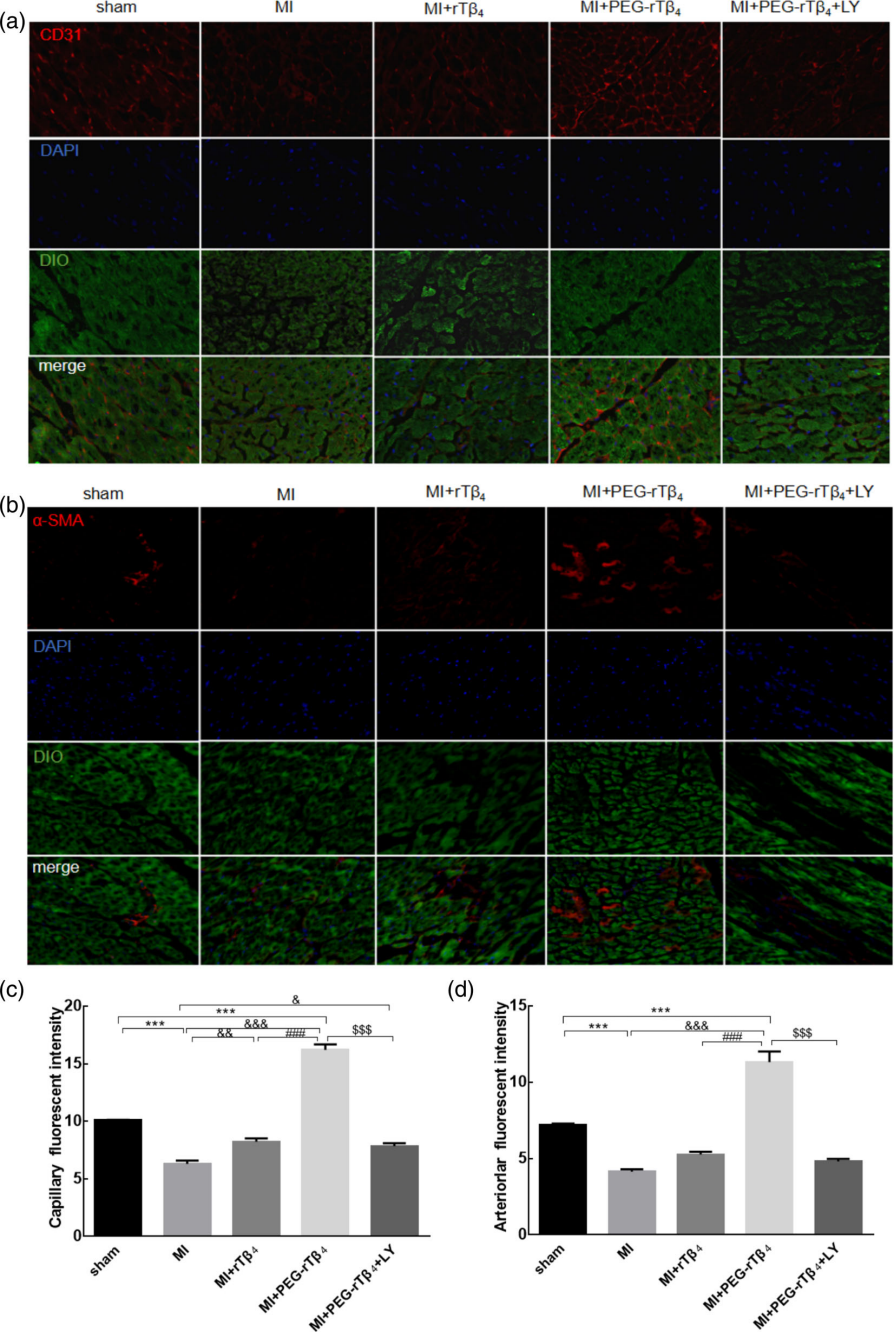
PEGylated recombination thymosin β‐4 (PEG‐rTβ_4_) accelerates angiogenesis in the infarct myocardium of myocardial infarction (MI) rats (*n* = 3). (a) and (b) show an image of platelet endothelial cell adhesion molecule 1 (PECAM 1) (CD31) and α‐smooth muscle actin (α‐SMA) immunofluorescent staining, respectively. (c) and (d) present the statistical analysis of (a) and (b), respectively. Statistical significance is indicated as follows: ***p < 0.001 for sham versus MI and sham versus MI + PEG‐rTβ_4_; ^&^ *p* < 0.05 for MI versus MI + PEG‐rTβ_4_ + LY294002 (LY); ^&&^ *p* < 0.01 for MI versus MI + rTβ_4_; ^&&&^ *p* < 0.001 for MI versus MI + PEG‐rTβ_4_; ^###^ *p* < 0.001 for MI + rTβ_4_ versus MI + PEG‐rTβ_4_; and ^$$$^ *p* < 0.001 for MI + PEG‐rTβ_4_ versus MI + PEG‐rTβ_4_ + LY. DAPI, 4′,6‐diamidino‐2‐phenylindole.

Ex vivo immunofluorescent staining was selected to evaluate neoangiogenesis. In Figure 7a,c, the group treated with PEG‐rTβ_4_ had higher myocardial capillary density than the other groups at 30 days post‐MI, as shown by CD31 immunostaining. The density of small functional arteries was tested by α‐SMA staining. As shown in Figure 7b,d, after the treatment of PEG‐rTβ_4_, the α‐SMA‐positive arterial density increased significantly compared to the treatment of rTβ_4_, indicating more vessel formation after MI. However, these phenomena did not occur in the MI and MI + PEG‐rTβ_4_ + LY groups.

**FIGURE 8:**
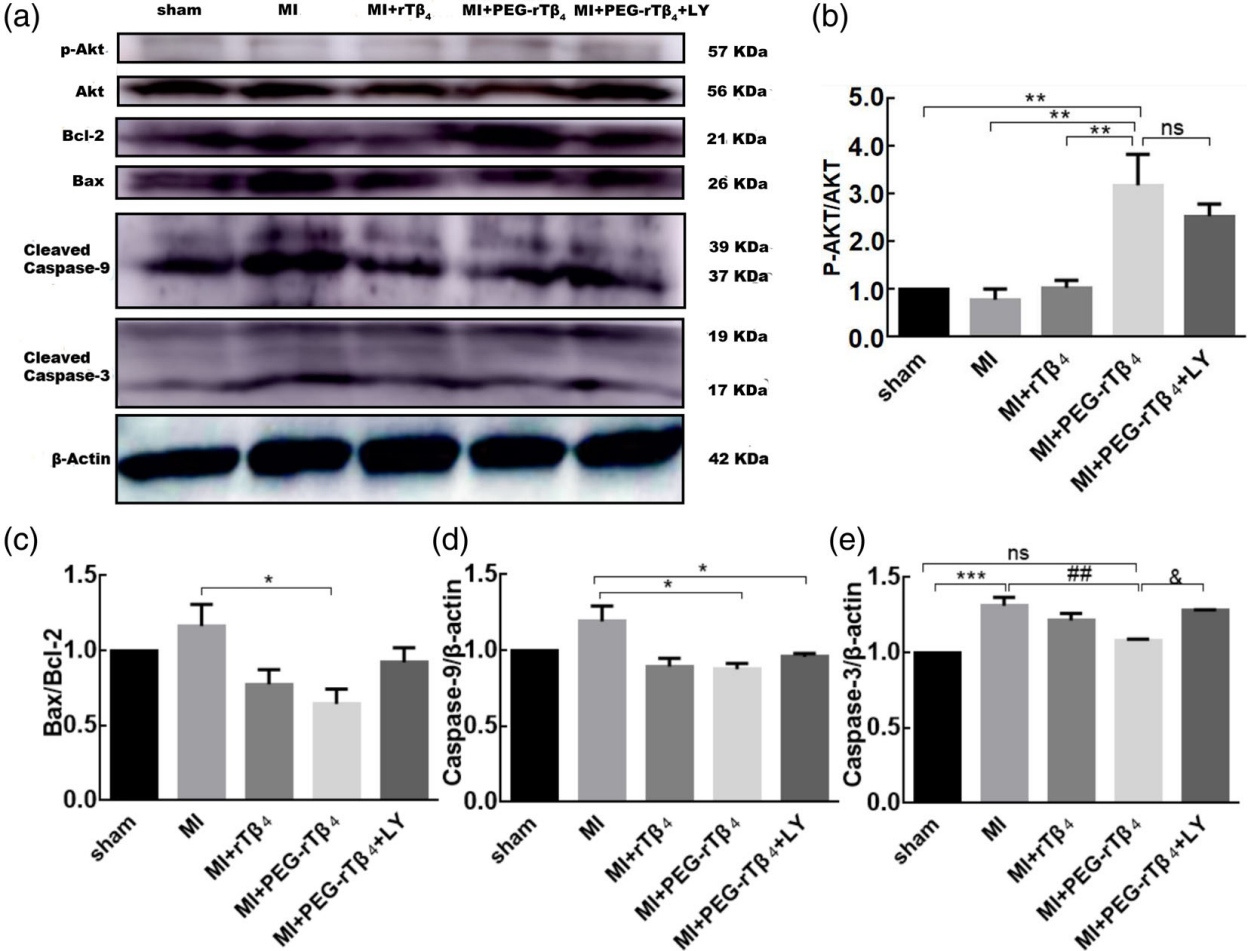
PEGylated recombination thymosin β‐4 (PEG‐rTβ_4_) inhibits cardiomyocyte apoptosis and preserves cardiac function via the Akt/Bcl‐2/caspase‐3 and caspase‐9 signaling pathway (*n* = 3). (a) Western blot analysis of Akt, p‐Akt, Bax, Bcl‐2, cleaved caspase‐3, and caspase‐9 in the ischemic penumbra region of the infarct myocardium. (b) Statistical analysis of the p‐Akt/Akt ratio. A statistically significant difference was observed (**p < 0.01) between myocardial infarction (MI) + PEG‐rTβ4 and sham, MI, or MI + rTβ4 groups; no significant difference (ns) was found between MI + PEG‐rTβ4 and MI + PEG‐rTβ4 + LY groups. (c) Statistical analysis of the Bax/Bcl‐2 ratio. A statistically significant difference was observed (*p < 0.05) between MI + PEG‐rTβ_4_ and MI groups. (d) Statistical analysis of caspase‐9 normalized to β‐actin. A statistically significant difference was observed (*p < 0.05) between MI and MI + PEG‐rTβ_4_ or MI + PEG‐rTβ_4_ + LY groups. (e) Statistical analysis of caspase‐3 normalized to β‐Actin. Significant difference was observed (***p < 0.001) between sham and MI groups; no significant difference (ns) between sham and MI + PEG‐rTβ_4_ groups; (^##^ *p* < 0.01) between MI and MI + PEG‐rTβ_4_ groups; and (^&^ *p* < 0.05) between MI + PEG‐rTβ_4_ and MI + PEG‐rTβ_4_ + LY groups.

At the histological level, PEG‐rTβ_4_ significantly improved ventricular remodeling and increased angiogenesis near the ischemic area after MI. We concluded that the inhibition of remodeling results from PEG‐rTβ4's anti‐apoptotic effect. At the same time, proangiogenesis may be caused by increased VEGF expression, an angiogenic cytokine, following Akt activation, as confirmed by immunohistochemistry (Figure 6b) and western blot (Figure 8a,b). Meanwhile, increased expression of CD31 on venules and of α‐SMA on small arteries was observed after histological analysis.

**FIGURE 9:**
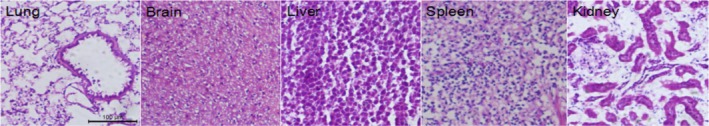
Hematoxylin/eosin staining of lung, brain, liver, spleen, and kidney from myocardial infarction rats after 4 weeks of treatment with PEGylated recombination thymosin β‐4.

In addition, HE staining of lung, brain, liver, spleen, and kidney tissue sections was performed. After 4 weeks of continuous PEG‐rTβ4 injection, no significant lesions were observed in these organs (Figure 9). Therefore, we firmly believe there are no secondary adverse reactions after long‐term continuous administration of PEG‐rTβ_4_.

### 3.6 PEG‐rTβ_4_ inhibits apoptosis via Akt/Bcl‐2/caspase‐3 signal pathway

Using data from apoptosis experiments in primary cardiomyocytes, we found that PEG‐rTβ4 significantly inhibited hypoxia‐induced cardiomyocyte apoptosis and reduced mitochondrial membrane damage. Furthermore, a previous study in MI rats showed that Tβ4 could target integrin‐linked kinase (ILK), particularly ILK‐partnered new Cys‐histidine‐rich protein 1 (PINCH‐1), to activate Akt and protect cardiac function after ischemic injury.^41^ Other studies of Tβ_4_ have shown that it can protect endothelial progenitor cells against apoptosis by down‐regulating caspase‐3 and caspase‐9.^39^ As we all know, the Bcl‐2 family regulates the mitochondrial pathway of cell apoptosis. Bcl‐2 is the critical downstream node of the Ak strain transforming/ Protein Kinase B signaling pathway.^42^

By analyzing cell apoptosis mechanisms and further exploring mitochondrial‐mediated apoptotic signaling pathways, we hypothesized that PEG‐rTβ_4_ acted through the same Akt/Bcl‐2/caspase‐3 signaling pathway to inhibit apoptosis in ischemic myocardium. This hypothesis has been supported by evidence in MI rats. Several critical mediators were evaluated for the anti‐apoptotic effects of the molecular signaling mechanisms induced by PEG‐rTβ_4_. Akt, p‐Akt, Bax, Bcl‐2, caspase‐3, and caspase‐9 were investigated for their well‐established roles in apoptosis. LY was used to inhibit Akt phosphorylation, inhibit cell proliferation, and promote cell apoptosis.^43^, ^44^ PEG‐rTβ_4_ accelerated Akt phosphorylation. At the same time, LY eliminated the effects of PEG‐rTβ_4_ on phosphorylated Akt (p‐Akt, Figure 8a). The phosphorylation rate (p‐Akt/Akt) in the MI + PEG‐rTβ_4_ group was approximately increased 2 or 2.5 times than sham or MI groups, respectively (Figure 8b). Moreover, PEG‐rTβ_4_‐increased phosphorylation rate may be caused by the transformation of Akt to p‐Akt, which indirectly reduces the amount of Akt; this also interprets that p‐Akt level was decreased because of LY while MI + PEG‐rTβ_4_ + LY group still in a higher phosphorylation rate.

Some proteins are closely related to apoptosis. Bcl‐2 can inhibit apoptosis and is an anti‐apoptotic protein. However, the effect of Bax is precisely the opposite, making it a pro‐apoptotic protein^45^ Bcl‐2 and Bax can form dimers; their ratio directly affects the degree of apoptosis.^45^, ^46^ After MI, Bax was significantly increased, but under the action of PEG‐rTβ4, Bcl‐2 was increased, directly reducing Bax (Figure 8a,c). The caspase‐3, as an upstream product of caspase‐9, increases earlier after a heart attack. The caspase‐3 and caspase‐9 were lowered by PEG‐rTβ_4_ when compared with other groups, and the results showed that there were no significant differences when compared to the sham group (Figure 8d,e); the failure of PEG‐rTβ_4_ in the signal pathway was caused by LY, which decreased p‐Akt, inhibited Bcl‐2, and increased caspase‐3 and caspase‐9.^47^, ^48^ The data also confirmed that PEGylation of rTβ_4_ did not alter the anti‐apoptotic mechanism of Tβ4, supporting the explanation provided by the DSC data.

### 3.7 Long circulation of PEG‐rTβ_4_ and rTβ_4_

**FIGURE 10:**
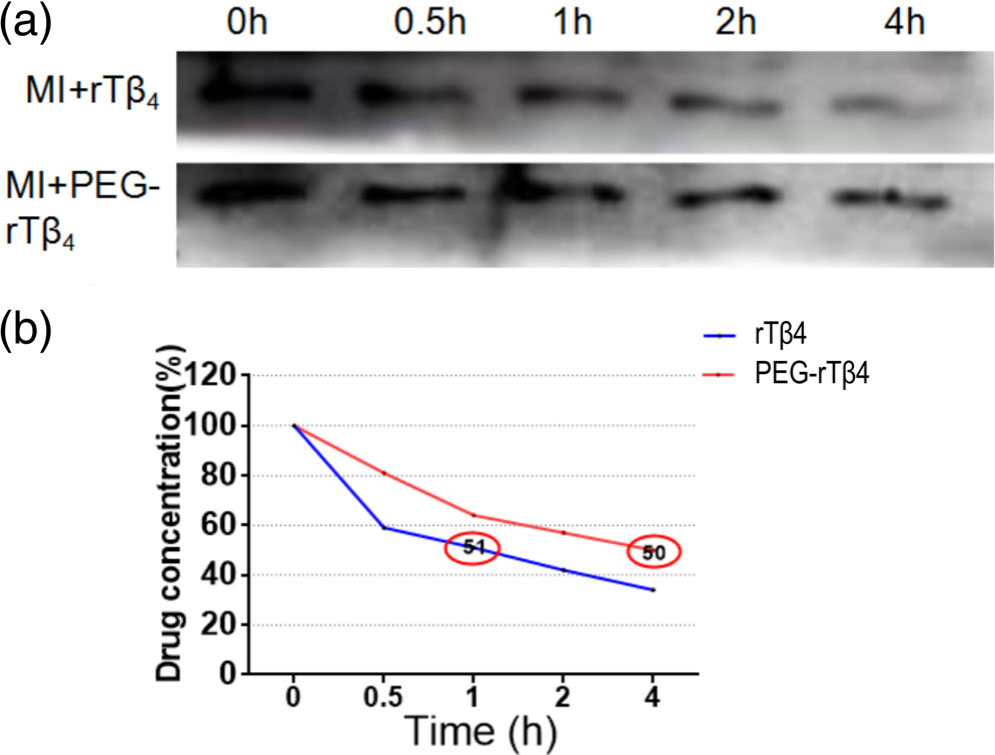
The pharmacokinetic analysis of PEGylated thymosin β‐4 (PEG‐rTβ_4_) and recombination thymosin β‐4 (rTβ_4_) in vivo (*n* = 6) was performed as follows: (a) After intravenous injection via the tail vein of PEG‐rTβ_4_ and rTβ_4_ into myocardial infarction (MI) rats, the levels of PEG‐rTβ_4_ and rTβ_4_ in serum decreased over time, as determined by western blot. (b) The percentage of remaining protein in serum relative to the initial administered dose (PEG‐rTβ_4_ shown by the red line and rTβ_4_ by the blue line) was measured at 0, 0.5, 1, 2, and 4 h post‐administration. The points corresponding to 50% and 51% remaining protein were marked with red circles that intersected the red and blue lines, respectively, indicating the approximate half‐lives of PEG‐rTβ_4_ and rTβ_4_.

Serum drug concentration of PEG‐rTβ_4_ and rTβ_4_ were determined by western blot (Figure 10a). The drug concentration curves showed that the drug half‐life of rTβ_4_ and PEG‐rTβ_4_ is about 1 and 4 h in MI rats, respectively (Figure 10b). Compared with Ruff's data, the rats were injected with 2 mg/kg of rTβ4, whose half‐life was about 1 h and similar to that of a healthy volunteer injected with Tβ_4_ of 42 mg/kg (0.95 h). In this study, animals were injected with 2.4 mg/kg of PEG‐rTβ_4_ (equivalent to 2 mg/kg of Tβ_4_), and its half‐life was approximately twice that observed in healthy volunteers injected with 1260 mg/kg of Tβ_4_.^49^ When the healthy volunteers' dose increased from 42 to 1260 mg/kg (30‐folds), the half‐life only doubled (0.95 vs. 2.1 h). These results indicate that the in vivo half‐life of rTβ_4_ was extended approximately four‐fold by PEG modification. This extension may explain why PEG‐rTβ_4_ was more effective than rTβ_4_ in reducing fibrosis, promoting angiogenesis, and inhibiting apoptosis. Extending a peptide's half‐life through PEGylation, thereby improving clinical efficacy and safety, is more meaningful than simply increasing the dosage. PEGylation to extend the peptide's half‐life, as well as its clinical efficacy and safety, is more meaningful than a dosage increase. PEGylation improved the utilization efficiency of rTβ_4_, thereby reducing medication costs in future clinical applications.

## 4 CONCLUSION

The present study provides convincing evidence that PEG‐rTβ_4_, a novel long‐circulating prodrug, provides significant cardioprotective effects in MI. It benefits by inducing anti‐apoptosis and neovascularization via the Akt/Bcl‐2/caspase‐3 signaling pathway in MI rats. These data support PEG‐rTβ_4_ as an excellent candidate drug for treating MI. Additionally, clinically relevant studies should be conducted soon.

## AUTHOR CONTRIBUTIONS

Conceptualization and design: **HP**, **QW**; Methodology: **YC**, **CG**, **YP**, **XL**, and **ST**; Data analysis and curation: **YH**, **YP**, and **WZ**; Investigation and validation: **XL**, **QK**; Writing: **YC**, and **CG**, **QW**; Supervision and funding acquisition: **HP**.

## CONFLICT OF INTEREST STATEMENT

The authors report no conflicts of interest. The authors alone are responsible for the content and writing of this article.

## Supporting information


**Data S1.** Supplementary data of rTβ_4_ and PEG‐rTβ_4_ include Western blot analysis, MALDI‐TOF mass spectrometry, molecular weight, and evaluation of experimental MI rats after ligation of the left anterior‐descending artery (LAD).

## Data Availability

These research data are openly available in a public repository that issues DOIs for data sets. The data supporting the findings of this study are openly available on Wiley and its repositories and will be freely distributed to qualified academic investigators for non‐commercial research upon request.
