# Supplementary material for: PEGylated thymosin β4 is a thiol‐site‐specific prodrug treating myocardial infarction in vivo
Source: Bioeng Transl Med. 2026 Mar 31;11(4):e70144. doi: 10.1002/btm2.70144 (PMC13327619; doi:10.1002/btm2.70144)
Supplement: Supplementary file 1 — Data S1. Supplementary data of rTβ4 and PEG‐rTβ4 include Western blot analysis, MALDI‐TOF mass spectrometry, molecular weight, and evaluation of experimental MI rats after ligation of the left anterior‐descending artery (LAD). [file BTM2-11-e70144-s001.docx]

**Supplementary Materials**


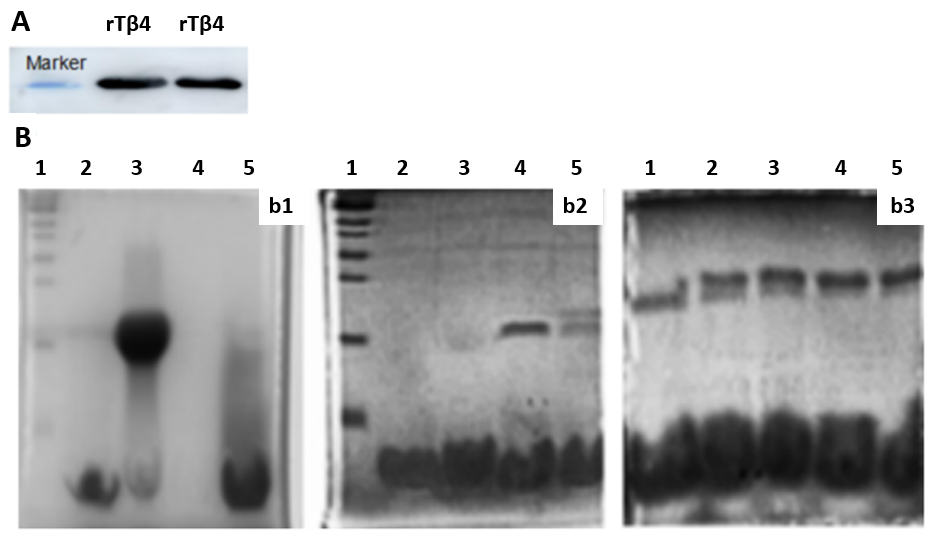


**Supplementary Fig. 1**. The identifies of rTβ_4_ and PEG-rTβ_4_. A. Western blot analysis of rTβ_4_. B. The conjugation of PEG-MAL with rTβ_4_ was characterized by iodine staining (b1) and gel-staining (b2 and b3). b1 is the Iodochromogram of protein Marker (lane 1), the mixture of rTβ_4_ and PEG-MAL (lane 2), PEG-rTβ_4_ (lane 3), rTβ_4_ (lane 4), and PEG-MAL (lane 5); b2 is the Gel-staining chromatogram of protein Marker (lane 1), the loading buffer (lane 2), PEG-MAL (lane 3), rTβ_4_ (lane 4), and unpurified PEG-rTβ_4_ (lane 5); b3 is the Gel-staining chromogram of rTβ_4_ (Lane 1) and purified PEG-rTβ_4_ (lane 2-5).


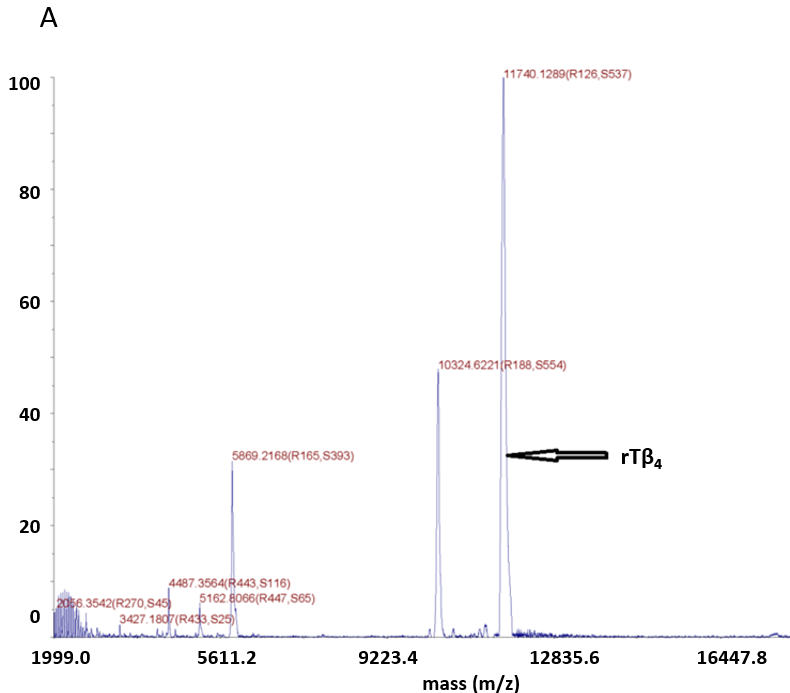


**Supplementary Fig. 2 A**. MALDI-TOF MS was used to identify rTβ_4_ and PEG-rTβ_4_. The molecular weight of rTβ_4_ is 11.74 KD.


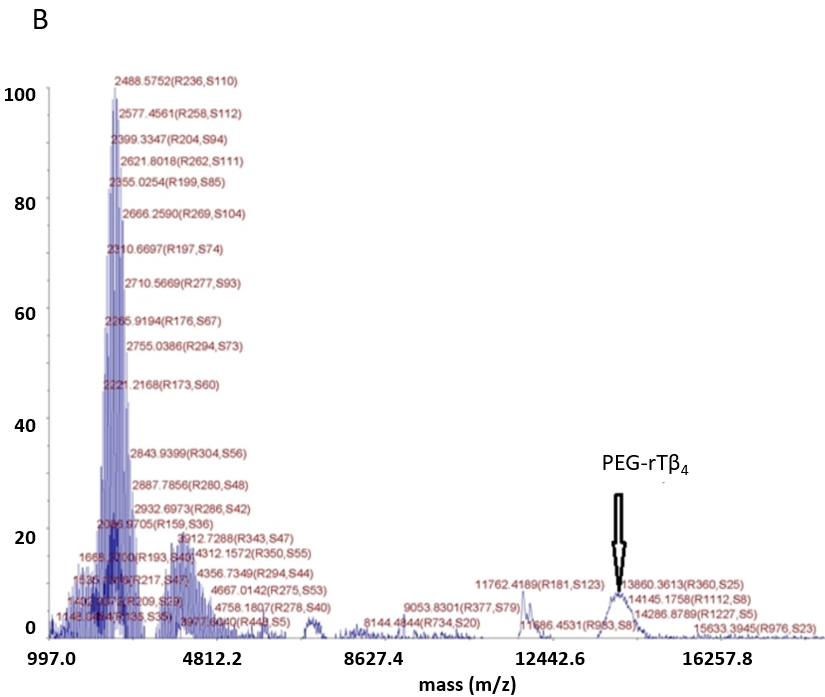


**Supplementary Fig. 2B**. Molecular weight of PEG-rTβ4 is 13.86 KD.


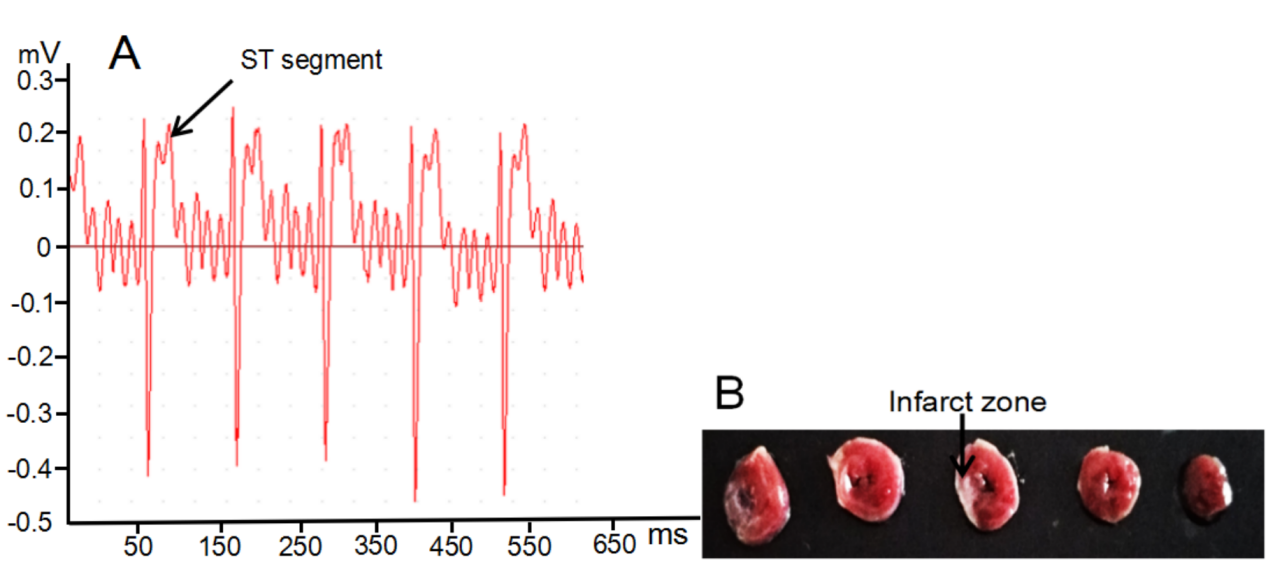


**Supplementary Fig. 3**. The evaluation of experimental MI rats after ligation of the left anterior descending artery (LAD). (A) The electrocardiograph of MI rats was recorded before and after the ligation of LAD. The black arrow points to the elevated ST segment. (B) After ligation of LAD, TTC staining was used to assess the degree of ischemia myocardium. The black arrow points to the white infarct zone.
